# PDGF-AA mediates mesenchymal stromal cell chemotaxis to the head and neck squamous cell carcinoma tumor microenvironment

**DOI:** 10.1186/s12967-016-1091-6

**Published:** 2016-12-08

**Authors:** Tammara L. Watts, Ruwen Cui, Peter Szaniszlo, Vicente A. Resto, Don W. Powell, Irina V. Pinchuk

**Affiliations:** 1 Department of Otolaryngology, University of Texas Medical Branch, 301 University Blvd, Galveston, TX 77555-0521 USA; 2 Internal Medicine, Division of Gastroenterology, University of Texas Medical Branch, Galveston, TX 77555 USA; 3 Microbiology and Immunology Department, University of Texas Medical Branch, Galveston, TX 77555 USA

**Keywords:** Tumor microenvironment, Head and neck cancer, Migration, Invasion, Mesenchymal stromal cells

## Abstract

**Background:**

The robust desmoplasia associated with head and neck squamous cell carcinoma (HNSCC) suggests that the tumor microenvironment may be an important component in the pathophysiology of this cancer. Moreover, the high recurrence rate and poor clinical response to chemotherapy and radiation treatment further underscores that the non-cancerous cells of the microenvironment, such as mesenchymal stromal cells (MSCs), cancer associated fibroblasts (CAFs), and pericytes, may be important in the pathophysiology of HNSCC.

**Methods:**

Confocal microscopy and immunohistomchemistry approaches were used to identify MSCs tumor microenvironment from patients with oral cavity and oral pharyngeal squamous cell carcinoma (SCC). In vitro Boyden chamber assays and multiplex magnetic bead assays were used to measure MSC chemotaxis and to identify the chemokines secreted by JHU-011, -012, -019, three cells lines derived from patients with oral pharyngeal SCC.

**Results:**

We show here that MSCs reside in the tumor microenvironment of patients with oral cavity and oral pharyngeal SCC and are recruited via paracrine mediated tumor cell secretion of (platelet derived growth factor) PDGF-AA. The MSC markers CD90^+^, CD105^+^, and gremlin-1^+^ were found to co-localize on cells within the tumor microenvironment in oral cavity SCC specimens distinct from α-smooth muscle actin staining CAFs. The conditioned media from JHU-011, -012, and -019 caused a significant increase in MSC migration (>60%) and invasion (>50%; p < 0.0001) compared to oral keratinocyte (OKT) controls. Tumor cell induced MSC chemotaxis appears to be mediated through paracrine secretion of PDGF-AA as inhibition of the PDGF-AA receptor, PDGFR-α but not PDGFR-β, resulted in near arrest of MSC chemotaxis (p < 0.0001).

**Conclusions:**

Tumor microenvironment expression of PDGFR-α has been shown to correlate with a worse prognosis in patients with prostate, breast, ovarian, non-small cell lung cancer and osteosarcoma. This is the first evidence that a similar signaling paradigm may be present in HNSCC. PDGFR-α inhibitors have not been studied as adjunctive treatment options in the management of HNSCC and may prove to be an important driver of the malignant phenotype in this setting.

**Electronic supplementary material:**

The online version of this article (doi:10.1186/s12967-016-1091-6) contains supplementary material, which is available to authorized users.

## Background

Head and neck squamous cell carcinoma (HNSCC) is the fifth most common cancer worldwide, the vast majority arising from the oral cavity (OC) and oropharynx (OP). Despite earlier detection rates, multimodality therapy and surgical advances, the overall 5 year survival rate for advanced HNSCC is poor (<25%), and has remained largely unchanged in the last 30 years. Local regional failure following attempts at curative treatment with either primary surgical excision and/or concurrent chemoradiation accounts for recurrence rates as high as 50%. The clinicopathologic response of HNSCC to conventional protocols including surgical excision and chemoradiotherapy suggests that treatment strategies directed toward tumor cells alone are inadequate and that targeting non-cancerous cells in the tumor microenvironment may improve clinical outcomes.

Mesenchymal stromal cells (MSCs), myofibroblasts/cancer associated fibroblasts (CAFs), pericytes, and other non-cancerous cells form a dense desmoplastic microenvironment around tumor cells and are known to be critical contributors to the growth of several solid tumors [1]. This rich desmoplastic reaction is a pathognomonic feature of HNSCC further driving the hypothesis that the tumor microenvironment is likely a key component in pathophysiology of this cancer. In addition to promoting cancer growth, MSCs and their differentiated progeny, CAFs, are known to confer resistance to chemotherapy and radiation, drive the development of cancer stem cells (CSC), and evade host immune responses [2]. The importance of the tumor microenvironment and its crosstalk with cancer cells are increasingly being recognized as important steps in the pathogenesis and progression of several cancers [1].

It is now believed that bone marrow derived MSCs serve a significant source of differentiated CAFs in the tumor microenvironment [2–4]. In models of gastric cancer, 20% of CAFs were shown to originate from the bone marrow and derived of MSCs [4]. In this model, bone marrow derived MSCs are thought to home to tumors via paracrine signals generated by the tumor itself [2, 5, 6], and once resident within the local microenvironment MSCs serve as important precursors for CAFs, together enhancing tumor growth through autocrine and paracrine signaling pathways [2, 7]. Co-culture of bone marrow derived MSCs, either direct or indirect via transwell culture, with breast cancer cells has been shown to significantly increase aldehyde dehydrogenase (ALDH) expression, a CSC marker [2]. The conditioned media alone from MSC was not enough to induce increased ALDH expression on the breast cancer cells, suggesting that the paracrine signaling feedback loop between the breast cancer cells and MSCs is necessary to drive the increase in ALDH expression [2].

Despite this recent knowledge in other epithelial cancer models, the role of MSCs, their localization within the tumor microenvironment of any HNSCC subsite including the OC and OP, and the signals governing MSC chemotaxis in this setting have not yet been described. Liotta et al. recently reported MSCs to be enriched in CD90^+^ stromal fraction of cells isolated from HNSCC tumors [8]. Prince et al. have also described isolation of ALDH^+^/CD44^+^ cells from patients with HNSCC are able to generate tumors de novo, and designated these as CSCs for their ability to recapitulate a heterogeneous tumor [9]. We report here that MSCs reside within the tumor microenvironment from patients with OC and OPSCC along with α-SMA^+^ CAFs. Moreover, HNSCC-induced migration of MSCs is driven by paracrine secretion of PDGF-AA.

## Methods

### Cell lines

Head and neck cancer cell lines JHU-011, JHU-012, JHU-019, JHU-022 (derived from human oropharyngeal tumors) and OKF-TERT1 human, immortalized oral keratinocyte cells (OKT) were generously provided by Dr. Vicente Resto, (Galveston, TX). Cells were maintained in RPMI 1640 medium containing glutamine supplemented with 10% fetal bovine serum at 37 °C in 5% CO_2_. Primary bone marrow-derived human mesenchymal stromal cells (MSCs) were obtained from Stem Cell Technologies (Vancouver, BC) and maintained according to the manufacturers recommendations. The human HNSCC cell lines used in these studies have been extensively characterized both in vitro and in vivo [10, 11].

### MSC migration and invasion

MSC chemotaxis was determined using the modified Boyden chamber assay, as previously described [12]. Briefly, 2.5 × 10^5^ MSCs in serum free media were seeded on the filter in the upper chamber of the transwell in a final volume of 250 µl. The lower chamber contained serum free control media, or conditioned media from JHU-011, -012, -019, -022 or OKT cells. The entire membrane was then analyzed and cells counted in five sections at 40×. MSC migration was quantified as a percentage increase or decrease normalized to OKT cell induced MSC migration. The average number of cells per membrane was determined that number, divided by the area of the microscope viewing field, and multiplied by the entire area of the transwell insert. All experiments were performed in triplicate for n ≥ 3. To determine chemoinvasion, transwells were prepared as described above and the filter plated with Matrigel™ (BD Biosciences, Franklin Lake, NJ) on the upper surface according to the manufacturer’s protocol.

### Histology and immunohistochemistry

Standard immunohistochemistry techniques were used as previously described on archival tissue from patients with HNSCC (Legacy Tumor Bank, Portland, Oregon) and the University of Texas Medical Branch, through an approved IRB study. Briefly, tissue sections of tongue and tonsil squamous cell carcinomas were deparaffinized and rehydrated, and antigen retrieval was performed in citrate-based antigen unmasking solution (Vector Laboratories, Burlingame, CA) at 90 °C for 20 min. Endogenous peroxidase was blocked using 3% hydrogen peroxide for 10 min. Nonspecific protein blocking was followed by incubation with primary and secondary antibodies and with ABC reagent, Vecstatin Elite ABC kit (Vector) was used following manufacturers recommendations. Isotype controls were incubated with rabbit IgG (Vector). ImmPact DAB (Vector) was used as the chromogen and hematoxylin (Sigma) as the counterstain. All stained sections were evaluated using a Leica DM LB microscope. Microphotographs were taken with a mounted Pixera PVC 100C camera. The percentage of gremlin-1 positive cells was measured using ImageJ and performing a threshold cutoff analysis. Experiments were performed in triplicate.

### Confocal microscopy

Confocal microscopy on frozen human oral cavity tissue sections (10 μm thickness) was performed as previously described with minor modifications [13]. Briefly, frozen human oral cavity tissue sections were fixed in 1% paraformaldehyde for 20 min at room temperature, blocked with normal murine serum (2.5% in PBS) for 15 min at room temperature, and then incubated with mix of AF^®^488-conjugated anti-α-SMA monoclonal Abs (0.2 µg/ml), AF^®^555 conjugated anti-human gremlin mouse mAbs (1 µg/ml) and/or AF^®^647 anti-human CD90 mouse mAbs for 1 h at room temperature. Each staining step was followed by six washes with PBS with Ca^++^/Mg^++^. Isotype controls were included in the analyses. The sections were then mounted in SlowFade^®^ Gold antifade reagent with DAPI (Life Technology, Inc.). Confocal microscopy was performed with a Zeiss LSM510 META laser-scanning confocal microscope (Carl Zeiss, Thornwood, NY) and confocal images analyzed using the LSM software and the following assignment of color: AF^®^-488 antibodies were depicted as green, AF^®^-555 antibodies were depicted as red, and AF^®^-647 depicted as blue. In merged images, co-localization of green and red antibodies resulted in yellow/orange image and co-localization of red and blue antibodies resulted in a magenta/pink image.

### Real-time RT-PCR

Real-time RT-PCR analysis was performed as previously described [14] according to the Applied Biosystems’s two-step RT real-time PCR protocol (Applied Biosystems, Foster City, CA). All reagents were purchased from Life Science Technology Inc. The appropriate assays-on-demand**™** gene expression assay mix (Applied Biosystems) for human actin RNA and the gene of interest (a 20× mix of unlabeled PCR primers and TaqMan^®^ MGB probe, FAM™ dye-labeled) and 2 µl of cDNA were added to the PCR reaction step. The reactions were carried out in a 20 µl final volume using a Bio-Rad Q5 real-time PCR machine according to the following protocol: 2 min at 50 °C, 10 min at 95 °C (1 cycle) and 15 s at 95 °C and 1 min at 60 °C (40 cycles).

### Western-immunoblotting

Standard Western blot analysis was performed on 10 μg of protein from the cell lines of interest using protocols as previously described [13]. PDGFRβ was purchased from Millipore (catalog # 06-495), PDGFRα (product # 9360) and GP130 from Cell Signaling Technology (product #3732), and α-IL6R from Thermo scientific (catalog #PA5-27975).

### Cytokine expression and production

Cytokine production was measured in the supernatants of the conditioned media from MSCs, primary fibroblasts-7049, OKT, and OPSCC JHU-011, -012, and -019 using appropriate cytokine ELISA kits (CXCL1, CXCL5, CXCL6, CXCL7); Milliplex kit (5-plex) IL6, IL8, VEGF, GRO, RANTES) (Millipore) and Bioplex human cytokine 21-plex, and 27-plex (Array kit; Bio-Rad) according to the manufacturer’s instructions. The Bio-Plex Array Reader and 96-well plate microplate platform were used as a reading system using xMAP detection technology (Bio-Rad).

### Statistical analysis

Unless otherwise indicated, the results were expressed as the mean ± SEM of data obtained from at least three independent experiments done with duplicate or triplicate sets. For nonparametric analysis differences between means were evaluated by two-way ANOVA. For parametric analysis paired (two-tailed) *t* test was performed. Values of *p* < 0.05 were considered statistically significant (Graphpad Prism 7.0).

## Results

### Mesenchymal stromal cells are identified within the OC and OPSCC tumor microenvironment

**Fig. 1:**
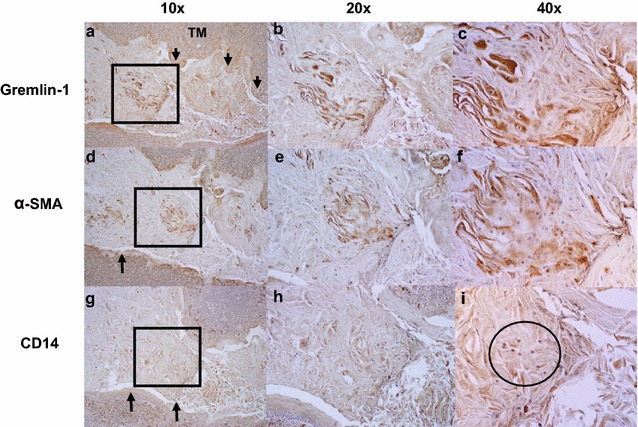
MSCs reside in the OPSCC (human tonsil) tumor stromal microenvironment. The *black arrows* denote the tumor microenvironment (TM). Representative immunohistological sections of human OPSCC demonstrates the presence of a distinct population of gremlin-1+ MSCs in the TM (**a**–**c**). The *black square* denotes areas viewed at higher magnification. Cells positive for the myofibroblast/CAF marker, α-SMA+ , were also present within the TM (**d**–**f**). A small population of CD14+ lymphocytes are seen at higher power magnification, representing cells of hematopoietic lineage (**g**–**i**). The above photomicrographs are serial sections (4 μm thickness)

Using an immunohistochemical approach, human OPSCC specimens (tonsil) were screened using the MSC marker gremlin-1. Gremlin-1, a more recently described MSC marker, is thought to specifically recognize peripherally migrating MSCs of bone marrow origin in models of gastric cancer [15]. Worthley et al. identified gremlin-1^+^ stromal cells co-localized with the epithelial stem cell niche within the gastrointestinal tract and specifically labeled mesenchymal cells that gives rise to α-smooth muscle actin (αSMA^+^) myofibroblast/CAFs [15, 16]. Representative immunostaining images from OPSCC tumor samples demonstrates the presence of gremlin-1 positive cells (Fig. 1a–c) in the tumor microenvironment from patient OPSCC tumor samples and labeled cells distinct from the hematopoietic lineage markers CD14 (Fig. 1g–i) and CD45 (Additional file 1: Figure S1A–C) an important distinction for determining cells of mesenchymal lineage. α-SMA, a mesenchymal lineage marker was also used to characterize the OPSCC tumor microenvironment. α-SMA + cells were seen in the tumor stroma and thought to represent myofibroblasts, which are cells thought to be progeny of MSCs (Fig. 1d–f). Approximately 18% of cells within the tumor microenvironment were noted to be gremlin-1 positive compared to IgG negative controls (Additional file 2: Figure S2).

**Fig. 2:**
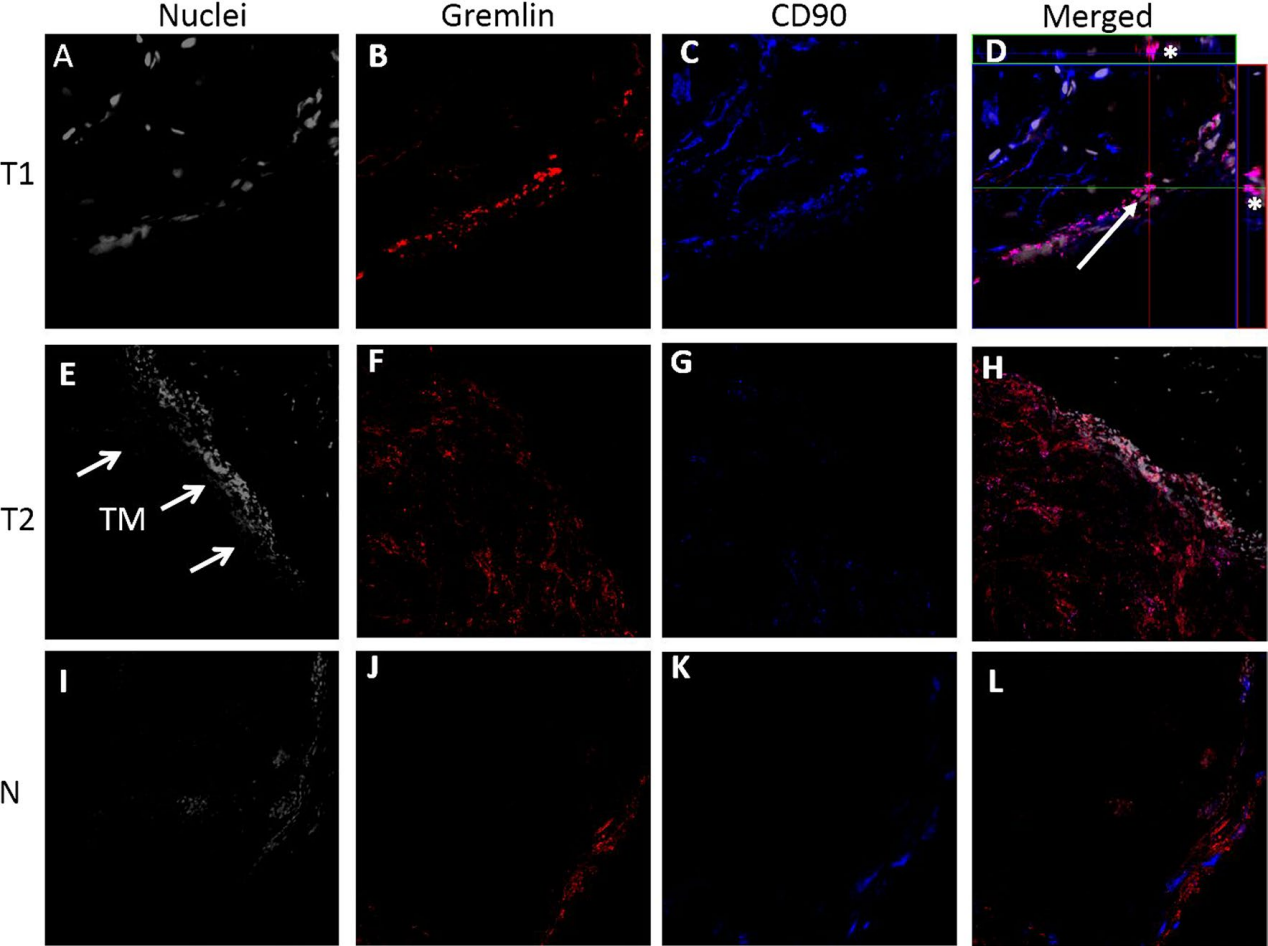
The MSC markers Gremlin-1 and CD90 are co-localized in the TM of patients with OCSCC (oral tongue). High power resolution confocal images from representative sections of 2 (T1 and T2) human OCSCC specimens (**A**–**H**) and adjacent normal (N) tissue (**I**–**L**) are shown. Gremlin-1+ cells (*red*; **A**, **F**) and CD90+ (*blue*; **C**, **G**) are identified in the TM and to a lesser extent adjacent normal tissue (**J**, **K**). When the photomicrographs are merged, co-localization of gremlin-1+ and CD90+ cells yields a magenta color (**D**, **H**). Additional, Z-stack image analysis with orthogonal sectioning (**D**, *white arrow*) demonstrates gremlin-1+/CD90+ cells (*magenta color*) in both the *x* and *y* planes

**Fig. 3:**
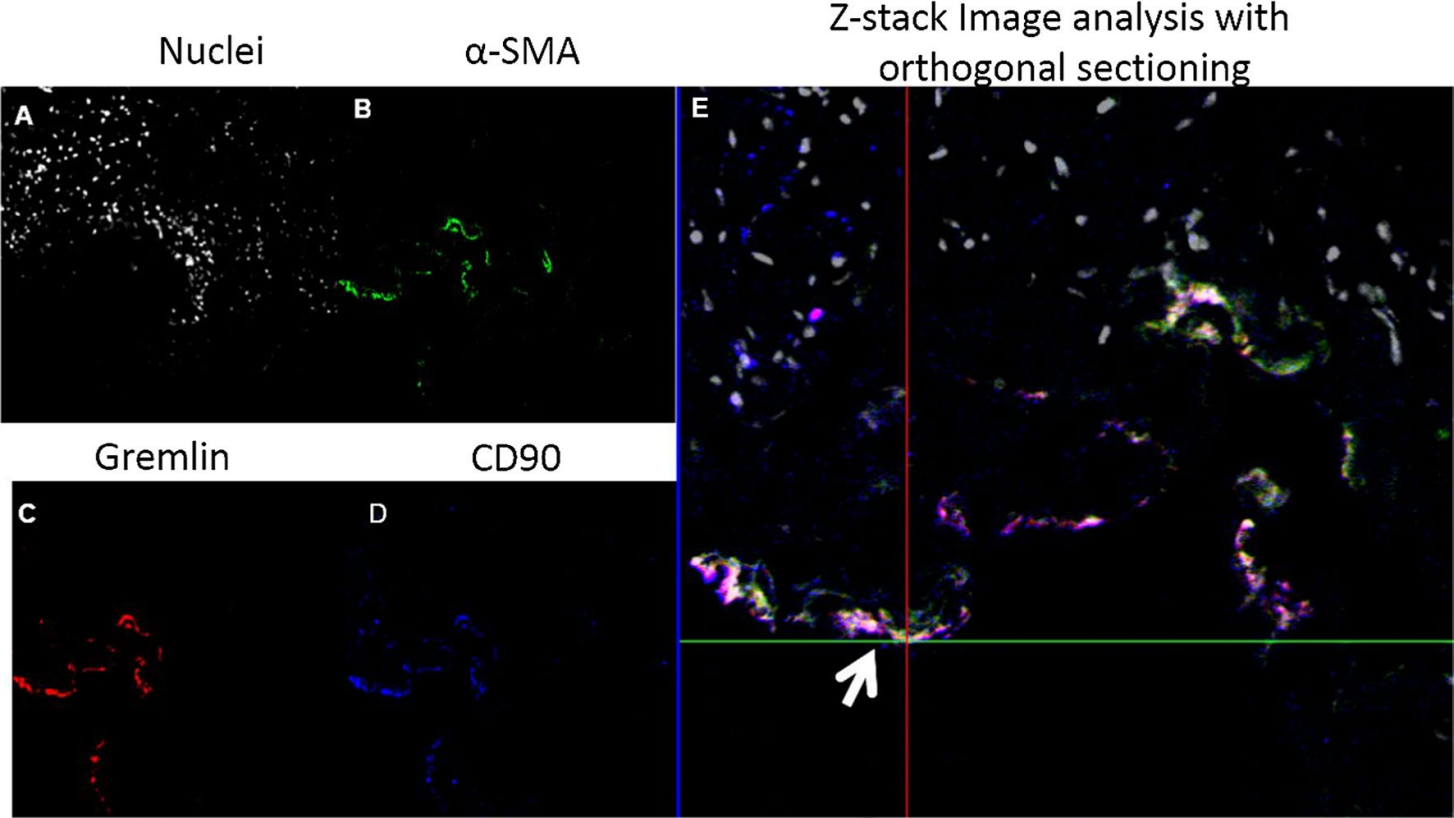
High resolution Z-stack confocal imaging OCSCC was performed to determine if α-SMA+ myofibroblasts (*green*; panel **B**) in the TM co-localized with the MSC markers Gremlin-1 (*red*; panel **C**) and CD90 (*blue*; panel **D**). On merged images (panel **D**), co-localization of gremlin-1 with CD90 yields a *magenta color* as indicated by the cross-hairs and *white arrow*. These cells are positive for the MSC markers gremlin and CD90 but not α-SMA. The nuclei are stained *grey* (panel **A**).

High resolution confocal microscopy demonstrates co-localization of the MSC markers gremlin-1 and CD90 of OCSCC (Fig. 2). Representative photomicrographs from 2/5 patient tumor samples and one adjacent normal control are shown. In Fig. 2, gremlin-1 immunostaining (B, F, and J) is denoted in red and CD90 immunostaining (C, G, and K) is denoted in blue. A magenta color signifies co-localization of gremlin-1 and CD90 on merged images (D, H, and L). Z-stack image analysis with orthogonal sectioning was performed on tumor specimen 1 (T1; Fig. 2D). At the cross-hairs (white arrow), cells are analyzed in both the x and y-planes, as denoted by the white asterisks, further supporting that the magenta color signal represents both co-localization of gremlin-1 and CD90 throughout the tissue slice. We also observed co-localization of gremlin-1 with the mesenchymal lineage marker CD105 (Additional file 3: Figure S4A) in the tumor stroma. Adjacent normal mucosa was observed to have relatively few MSCs compared to tumor specimens in 3/3 patient samples and a representative immunostaining shown in Fig. 2I–L). Further, the co-localization of gremlin-1^+^/CD90^+^ double positive cells with α-SMA^+^, which identifies differentiated myofibroblasts (known as CAFs in tumors) was limited (Fig. 3). Additionally, no co-localization with hematopoietic marker CD45^+^ cells were observed (Additional file 3: Figure S4B). Taken together these observations support our hypothesis that the gremlin-1^+^/CD90 +/CD105 + cells in the OC and OPSCC tumor microenvironment are MSCs.

### MSCs migrated toward OPSCC tumor cell condition media

**Fig. 4:**
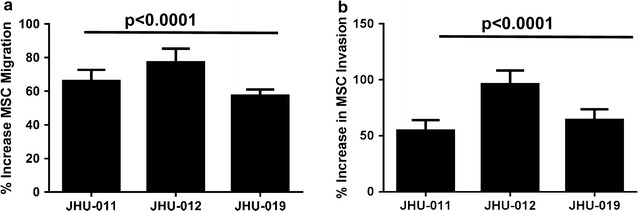
Conditioned media from 3 well characterized OPSCC cells lines (JHU-011, -012, and -019) caused significant migration and invasion of MSCs. Following 24-h incubation with conditioned media from JHU-011, -012, and -019, a >60% increase in MSC migration was observed when compared to OKT controls (**a**). MSCs were also observed to have a significant increased capacity for invasion >50% compared to that caused by the conditioned media from OKT controls (**b**). All data are expressed as a percentage increase in MSC migration and invasion normalized to OKT controls. Each experiment was performed in triplicate for n ≥ 3 (p < 0.0001)

Having identified cells of bone marrow mesenchymal origin in the tumor microenvironment from patients with OC- and OPSCC, we next tested whether tumor cells secrete chemotactic factors capable of inducing MSC homing in this context, in vitro. The condition medium from 3 well characterized OPSCC cell lines (JHU-011, -012, and -019 [11]) were shown to cause a >60% increase in MSC migration (Fig. 4a) and a >50% increase in MSC invasion (Fig. 4b) compared to normal oral keratinocytes (OKT; p < 0.0001) and serum free controls (Additional file 4: Figure S5). These observations suggested that several HNSCC tumor cells produce soluble factor(s) that may be responsible for the migration of the MSC to the local microenvironment.

### PDGFs and IL-6 are major factors implicated in HNSCC-mediated MSC chemotaxis

**Fig. 5:**
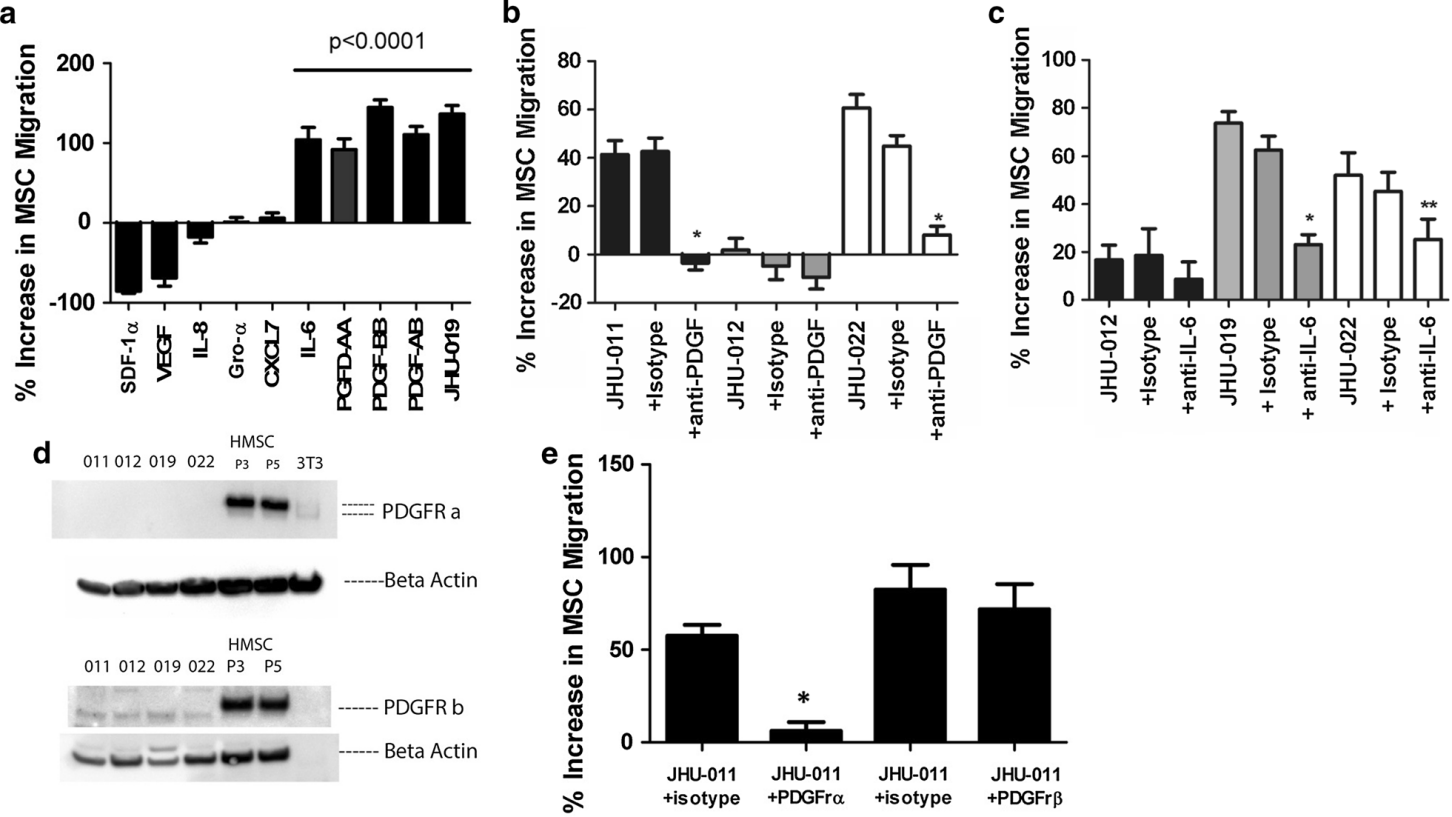
Seven chemokines and growth factors were noted to be highly secreted in the conditioned media of JHU-011 and JHU-019. Of those, only recombinant IL-6 and PDGF isoforms AA, BB, and AB independently caused significant migration of MSCs to levels comparable to OPSCC cell line JHU-019 when compared to 1% BSA treated media (**a**). Neutralizing antibodies to all PDGF isoforms (**b**) and IL-6 (**c**) in the conditioned media of cell lines JHU-011, -012, and -019 and/or -022 resulted in a significant reduction in MSC migration. PDGFRα and PDGFRβ was expressed on MSCs and not JHU-011, -012, -019 or JHU-022. 3T3 cells were used as a negative control (**d**). Only blockade of PDGFRα but not PDGFRβ resulted in a significant reduction in JHU-011 induced MSC chemotaxis. Two MSC passes P3 and P5 were used to ensure MSC passage did not affect PDGFR expression. Each experiment was performed in triplicate for n ≥ 3 and unless where indicated (*p < 0.0001, **p < 0.03)

Next, the conditioned media from OKTs, JHU-011, JHU-12 and -019 was analyzed for >50 chemokines and growth factors previously shown to be important in the pathophysiology of human cancer (Additional file 5: Figure S6). Eight growth factors and chemokines were identified as potential chemotactic factors (Additional file 6: Figure S7), of which IL-6, PDGF-AA, PDGF-BB and PDGF-AB independently caused a significant migration of MSCs comparable to that observed with JHU-019 (Fig. 5a, *p < 0.0001). Neutralizing antibodies were used to inhibit IL-6 or PDGF in the condition media to determine which chemokine was the primary driver of MSC chemotaxis in this setting. Neutralization of PDGF resulted in a >50% reduction in 3/3 HNSCC cell lines with a near complete arrest of MSC chemotaxis in 2/3 HNSCC cell lines (Fig. 5b; *p < 0.0001) whereas neutralization of IL-6 resulted in a ~50% reduction in MSC chemotaxis in 1/3 cell lines (Fig. 5c; *p < 0.0001, **p < 0.03). The negative migration response reflects a decrease in the percentage of MSC chemotaxis compared to OKT control cells. A similar response was observed when the IL6 and PDGF receptors were blocked in the upper chamber of the transwells (data not shown). Expression of PDGFRα and PDGFRβ was found to be exclusively present on the membrane of MSCs and not HNSCC cells lines JHU-011, -012, -019 or -022 (Fig. 5d). However, the IL-6 receptor and the IL-6 transducer, gp130, were found on the surface of both HNSCC cancer cells and MSCs (Additional file 7: Figure S8). Blockade of PDGFRα resulted in a significant reduction of MSC chemotaxis whereas blockade of PDGFRβ did not (Fig. 5e; p < 0.0001).

**Fig. 6:**
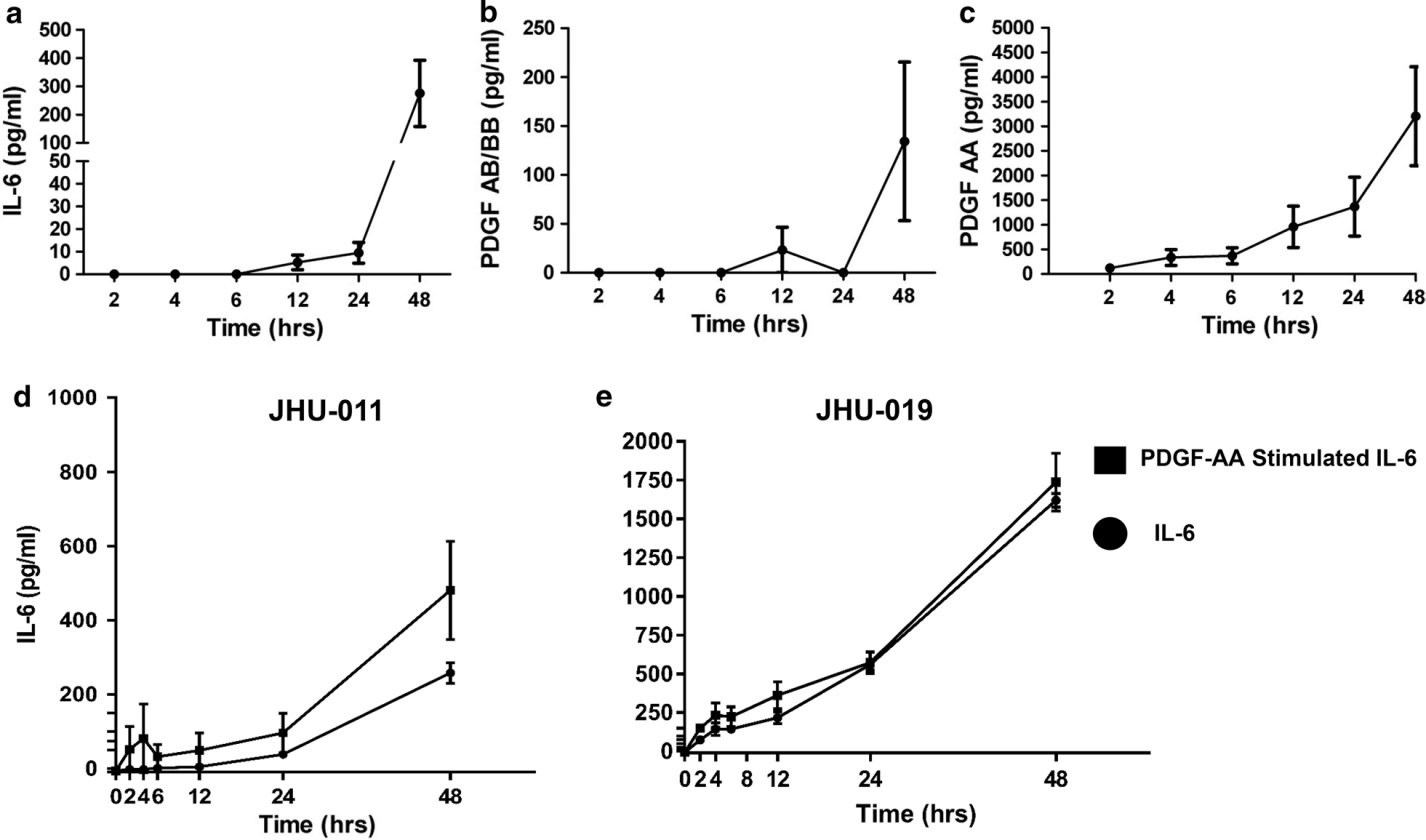
Secretion of PDGF-AA (**a**), PDGF-BB/AB (**b**), and IL-6 (**c**) were quantified over time using a magnetic bead assay approach. Concentrations of PDGF-AA rose earlier and were significantly higher than PDGF-BB, -AB or IL-6 in the conditioned media of JHU-011 and -019 (p < 0.007). To determine if PDGF-AA mediated secretion of IL-6, JHU-011 and -019 cells were treated with 5 ng PGGF-AA and IL-6 secretion compared to unstimulated cells (**d**, **e**). Stimulation with PDGF-AA resulted in a significant increase in IL-6 secretion (p < 0.001). All experiments performed in duplicate for n = 2 observations

To better understand if PDGF secretion by HNSCC tumor cells was dependent upon IL-6 or vice versa a multiplex bead assay approach was used to determine the kinetic secretory prolife. In both JHU-011 and -019 cell lines, PDGF-AA was found to be secreted early and at significantly high levels compared to either PGDF-AB, -BB, or IL-6 (Fig. 6a–c, p < 0.007). After 48-h, PDGF-AA secretion remained elevated without evidence of reaching a plateau, suggesting, PDGF-AA is likely the key chemotactic agent underlying MSC chemotaxis in HNSCC and may cause tumor cell release of IL-6 as a later phenomenon. Indeed, stimulation of JHU-011 and -019 with PDGF-AA resulted in a significant increase in IL-6 production compared to unstimulated cells, suggesting the PDGF-AA mediates release of IL-6 in this setting (Fig. 6d–f). PDGF-AA secretion by HNSCC appears to function in a paracrine fashion, as PDGF-AA exclusively signals through the PDGFα receptor, which was present in MSCs, but absent in JHU-011, -12, or -019 (Fig. 5d).

## Discussion

The overall survival rate for advanced head and neck squamous carcinoma cell (HNSCC) is dismal (<25%), and these statistics have remained largely unchanged for decades despite advances in surgical technique and multimodality therapy. This low survival and absence of efficient therapies exist because of a gap in knowledge about pathophysiology of the HNSCC tumor microenvironment giving rise to an unmet need in the >500,000 patients diagnosed annually with HNSCC. MSCs have been shown to home early to the tumor microenvironment of several columnar, epithelial, and CNS cancers (breast [7, 17], ovarian [6], gastric [4], melanoma [12], glioma [18]) and evade host immune responses [19]. The vast majority OC and OPSCC arises in the setting of long standing tobacco and/or alcohol abuse, resulting in a chronic state of inflammation [20]. MSCs have been shown to migrate in response to chemotactic stimuli secreted during tissue injury, inflammation, and cancer [6, 21]. Therefore, the presence of MSCs in microenvironment of OC and OPSCC is likely. However, studies demonstrating the presence of MSCs in the HNSCC tumor microenvironment are just emerging. Liotta et al. recently reported MSCs to be enriched in CD90^+^ stromal fraction of cells isolated from HNSCC tumors [8]. We have expanded on this emerging concept and demonstrate the presence of gremlin-1 +/CD90 +/CD105 + MSCs in tumor microenvironment of patients with OC and OPSCC (Figs. 1, 2, 3).

MSC mediated chemotaxis to the HNSCC tumor microenvironment is dependent upon tumor cell secretion of PDGF-AA and this mechanism of tumor driven recruitment of stem/stromal cells to the microenvironment has not been previously reported in HNSCC. Tumor microenvironment expression of PDGFR-α has been shown to correlate with a worse prognosis in patients with prostate, breast, ovarian, non-small cell lung cancer and osteosarcoma.

The PDGF-AA isoform is an important regulatory molecule in cell migration, wound healing that is thought to be among the most potent stimuli for cells of mesenchymal origin. This highly specific PDGF isoform signals only through the PDGF-α receptor which was not present on the surface of our OPSCC cells, suggesting a paracrine signaling mechanism is operate in OPSCC. We have shown here that PDGF-AA causes a ~100% increase in the migration of HMSCs compared to OKT cells and neutralization of PDGF in the conditioned media from OPSCC cell lines resulted in arrest of MSC chemotaxis to levels comparable with controls (Fig. 5b). Neutralization of IL-6 resulted in reduced HNSCC-induced HMSC chemotaxis (Fig. 5c), however the net reduction observed was less than that with PDGF inhibition alone. Our hypothesis that PDGF-AA is the primary driver of MSC chemotaxis in OPSCC is further strengthened as inhibition of only the PDGFRα significantly reduced MSC migration (Fig. 6e). Although both PDGFRα and PDGFRβ were found on the surface of MSCs, PDGF-AA only activates the PDGFRα not PDGFRβ, and inhibition of PDGFRβ did not cause the same magnitude of reduced chemotaxis as inhibition of PDGFRα.

PDGFR-mediated signaling pathways are highly associated with cancer progression. PDGFRα and PDGFRβ expression on breast cancer and the surrounding tumor stroma has been shown to be highly correlated with tumor aggressiveness and metastasis, respectively [22]. Imatinib, a selective inhibitor of PDGFR, was found to impair the tumor promoting effects of bone marrow derived MSCs in an orthotopic model of colon cancer [23]. Chayama et al. found cultured KM12SM colon cancer cells secreted PDGFβ but lacked expression of PDGFRβ [24]. Treatment with imatinib inhibited MSC migration, and significantly inhibited the tumor-promoting effects of MSCs and reduced the formation of liver metastasis. Furthermore, paracrine activation of PDGFRs has previously been reported to be important for the development of the surrounding tumor stroma which is largely thought to be derived from differentiated MSCs [22].

We have observed a similar paracrine activation signaling in OPSCC through tumor cell secretion of PDGF-AA. Our data suggest that PDGF-AA is a more potent MSC chemoattractant in OC and OPSCC, than other PDGF isoforms and IL-6. Previous reports have shown that the PDGF-AA, -AB, and -BB stimulates the transcription of the IL-6 gene in normal, nontransformed fibroblasts, vascular smooth muscle cells and mesangial cells [25]. Moreover, activation of the IL-6 gene was shown to serve as a key mechanism for proliferation of these cells of mesenchymal origin [31]. IL-6 working in an autocrine feedback loop on tumor cells and MSCs may further drive proliferation in this setting. The relationship and biologic significance between PDGF and IL-6 in the setting of OC and OPSCC is under active investigation in our lab.

## Conclusions

Our data suggests that HNSCC tumor cells production of PDGF-AA and IL-6 may be responsible to the increased tropism of MSC to the tumor site, which lays the foundation for further study in the MSCs in this context and the development of novel cellular/chemokines targeting therapies for this cancer.

## Additional files

**Additional file 1: Figure S1.** CD45 + lymphocytes within the tumor microenvironment of OPSCC. A population of CD14 + lymphocytes are seen representing cells of hematopoietic lineage (A-C). The black box denotes areas visualized under higher power magnification. The above photomicrographs are serial sectios (4 μm thickness).

**Additional file 2: Figure S2.** IgG negative controls at 20× magnification. The above photomicrographs are serial sections (4 μm thickness).

**Additional file 3: Figure S4.** The MSC markers gremlin-1 and CD105 are co-localized in the TM of patient with OCSCC (oral tongue). High power resolution confocal images from representative sections of human OCSCC specimens (A) demonstrate TM cells positive for the MSC markers anti-gremlin-1 (red) and anti-CD105 (green). An orange color on merged images indicates co-localization of gremlin-1 and CD105. In addition, TM MSCs were detected by anti-gremlin-1 (red) and anti-CD90 (blue). A magenta color on merged images indicates co-localization of gremlin and CD90 that did not co-localize with the hematopoietic marker, anti-CD45 (green) (B). The nuclei are depicted as grey.

**Additional file 4: Figure S5.** Conditioned media from 3 well characterized OPSCC cells lines (JHU-011, -012, and -019) caused significant migration and invasion of MSCs compared to serum free controls. Following 24-hour incubation with conditioned media from JHU-011, -012, and -019, a > sevenfold increase in MSC migration was observed when compared to serum free controls (A). MSCs were also observed to have a significant increased capacity for invasion > tenfold compared to that caused by the serum free controls (B).

**Additional file 5: Figure S6.** Preliminary screening of condition media from OKT, JHU-011, -012, and -019 was performed using multi bead 21- and 27-plex assay from Bio-Rad. From this screening, SDF-1α, VEGF, Gro-α, IL-8, IL-6, and PDGF were found to be highly secreted compared to the conditioned media from OKT controls. Experiments were performed in duplicate for n = 3 observations.

**Additional file 6: Figure S7.** Using the preliminary screening data, ELISA assays were conducted on SDF-1α, VEGF, Gro-α, IL-8, IL-6, and PDGF with the addition of CXCL7 to confirm the observations made in Additional file 1: Figure S6.

**Additional file 7: Figure S8.** The IL-6 receptor and gp130 was expressed on the surface of OPSCC JHU-011, -012, -019 and on MSCs. Experiments were performed in triplicate.

## Abbreviations

MSCs: mesenchymal stromal cells
CSCs: cancer stem cells
HNSCC: head and neck squamous cell carcinoma
OCSCC: oral cavity squamous cell carcinoma
OPSCC: oropharyngeal squamous cell carcinoma
ALDH: PDGF-AA, PDGFR-α, PDGFR-β, aldehyde dehydrogenase
TM: tumor microenvironment
