# Supplementary figures and images for: PDGF-AA mediates mesenchymal stromal cell chemotaxis to the head and neck squamous cell carcinoma tumor microenvironment
Source: J Transl Med. 2016 Dec 8;14:337. doi: 10.1186/s12967-016-1091-6 (PMC5146849; doi:10.1186/s12967-016-1091-6)

## Slide 1
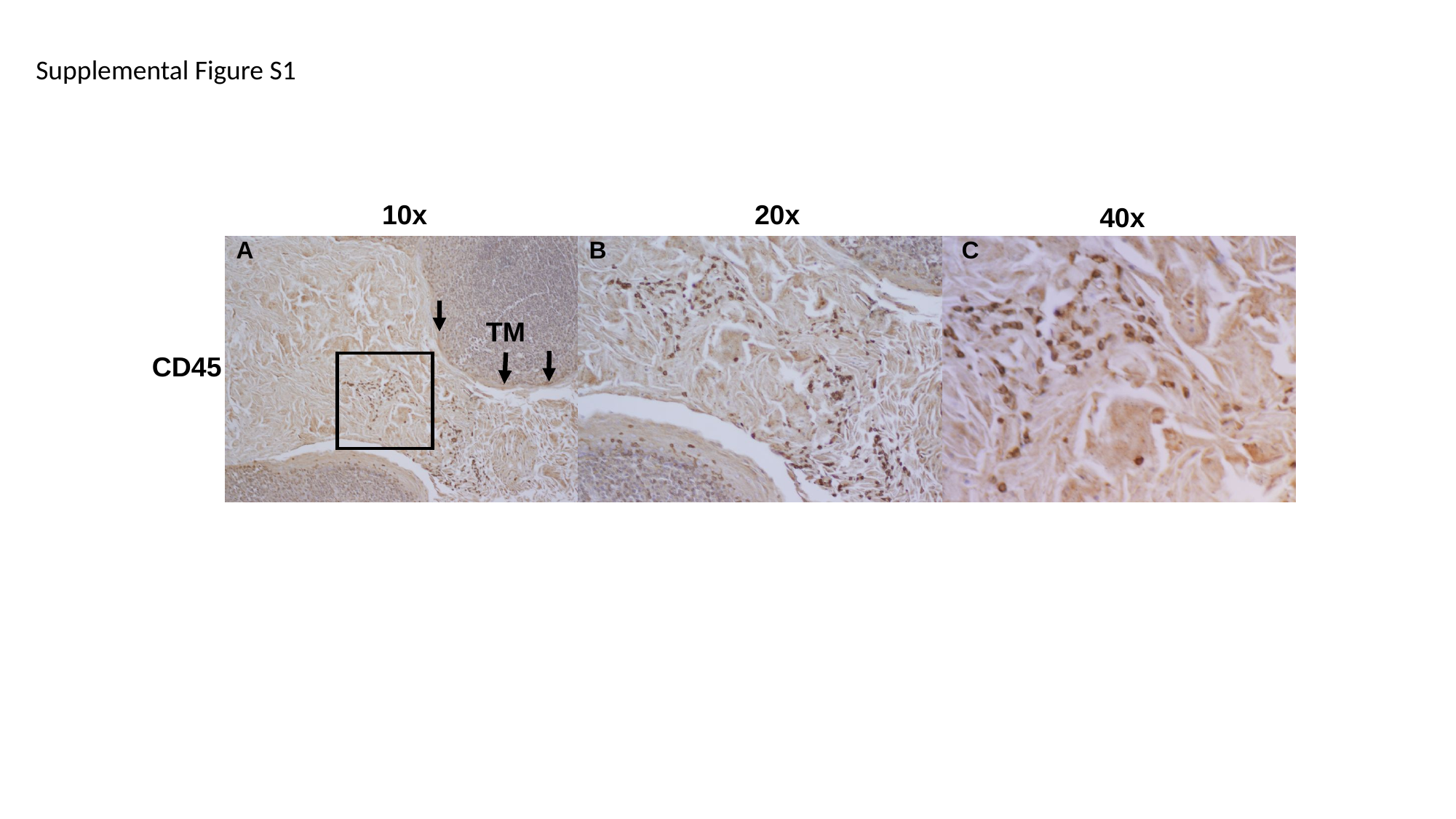

Supplemental Figure S1
10x
20x
40x
A
B
C
TM
CD45

Supplement: Supplementary file 1 — Additional file 1: Figure S1. CD45 + lymphocytes within the tumor microenvironment of OPSCC. A population of CD14 + lymphocytes are seen representing cells of hematopoietic lineage (A-C). The black box denotes areas visualized under higher power magnification. The above photomicrographs are serial sectios (4 μm thickness). [file 12967_2016_1091_MOESM1_ESM.pptx]

## Slide 1
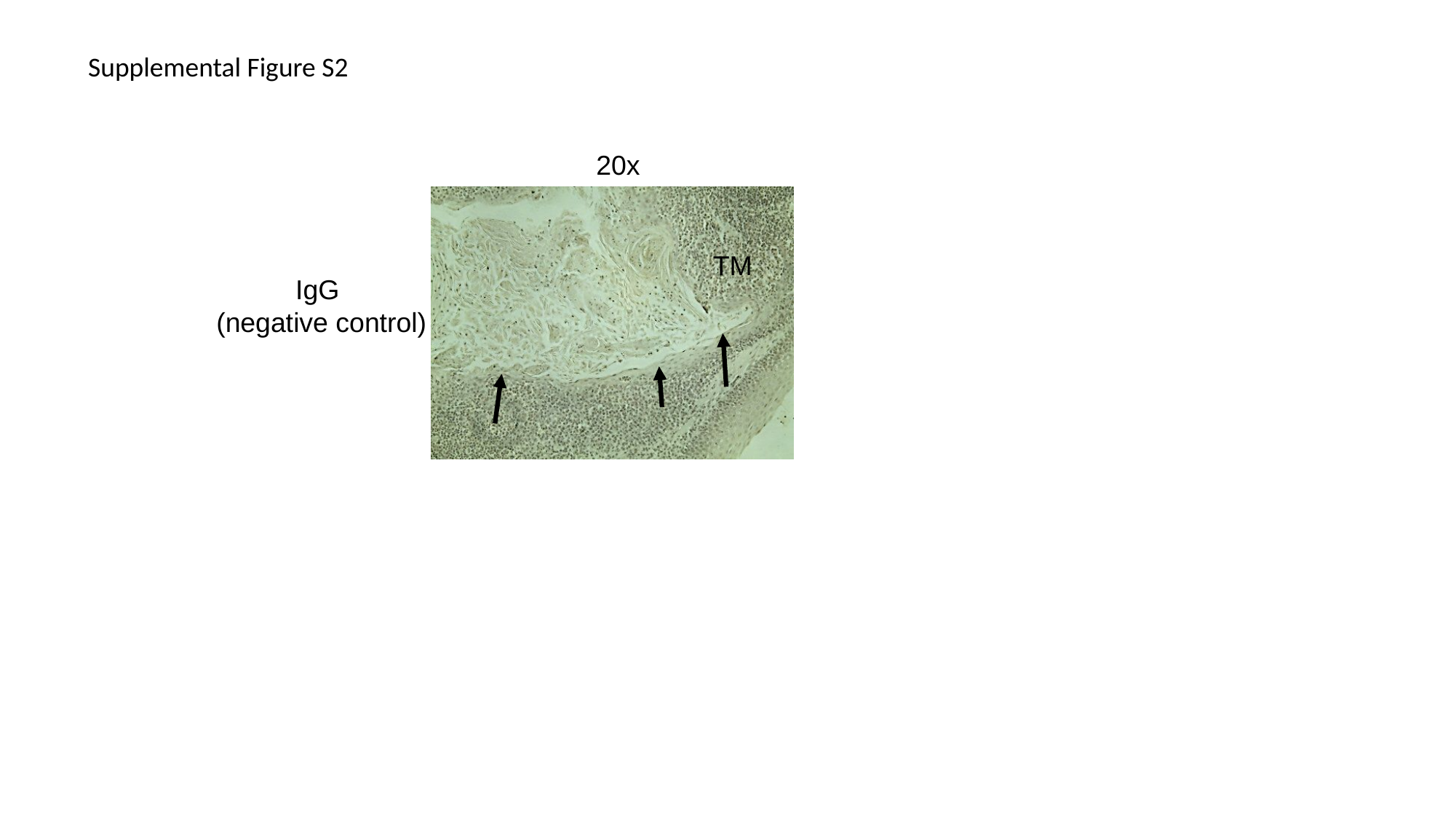

Supplemental Figure S2
20x
TM
IgG
(negative control)

Supplement: Supplementary file 2 — Additional file 2: Figure S2. IgG negative controls at 20× magnification. The above photomicrographs are serial sections (4 μm thickness). [file 12967_2016_1091_MOESM2_ESM.pptx]

## Slide 1
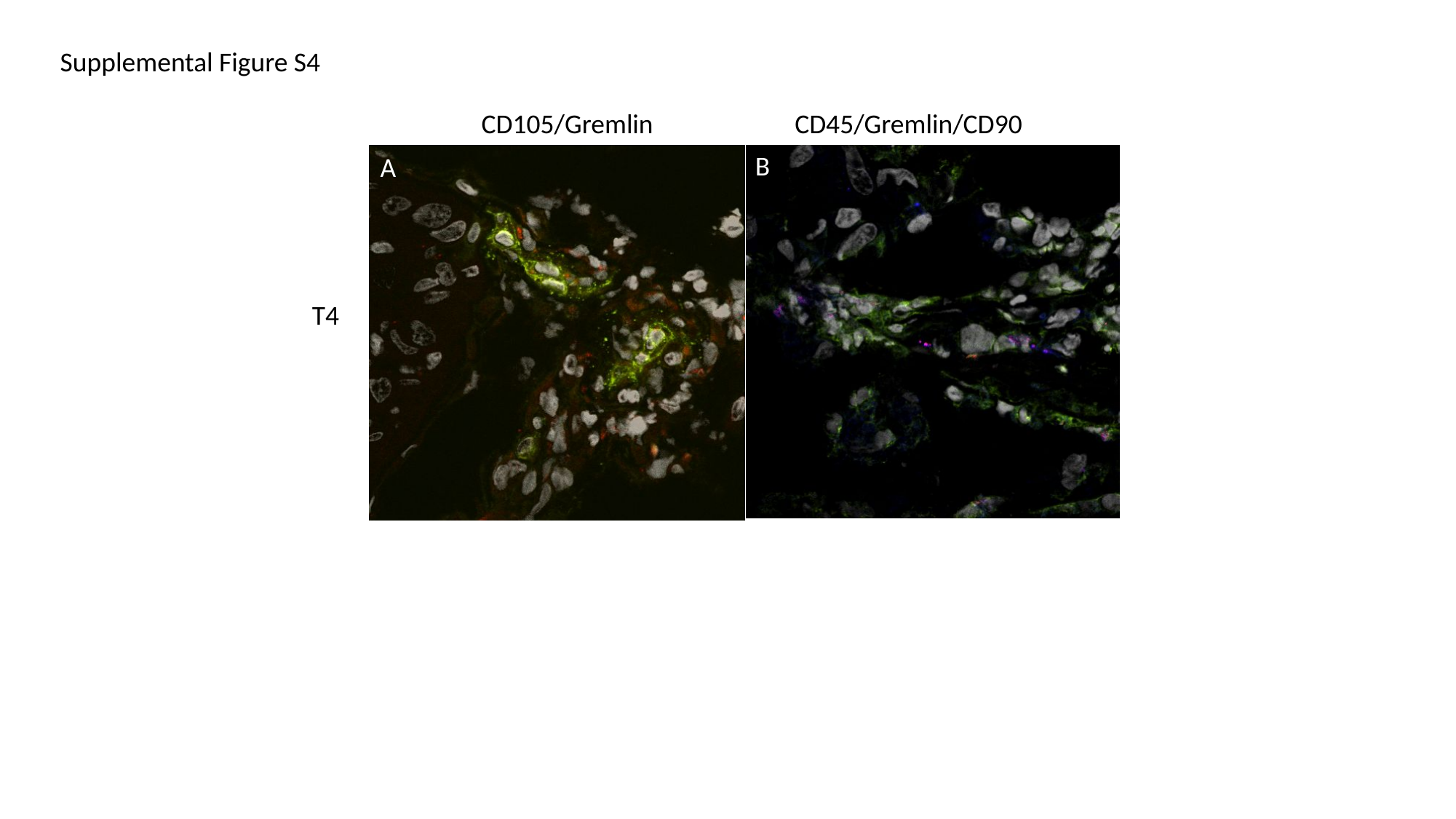

Supplemental Figure S4
CD105/Gremlin
CD45/Gremlin/CD90
B
A
T4

Supplement: Supplementary file 3 — Additional file 3: Figure S4. The MSC markers gremlin-1 and CD105 are co-localized in the TM of patient with OCSCC (oral tongue). High power resolution confocal images from representative sections of human OCSCC specimens (A) demonstrate TM cells positive for the MSC markers anti-gremlin-1 (red) and anti-CD105 (green). An orange color on merged images indicates co-localization of gremlin-1 and CD105. In addition, TM MSCs were detected by anti-gremlin-1 (red) and anti-CD90 (blue). A magenta color on merged images indicates co-localization of gremlin and CD90 that did not co-localize with the hematopoietic marker, anti-CD45 (green) (B). The nuclei are depicted as grey. [file 12967_2016_1091_MOESM3_ESM.pptx]

## Slide 1
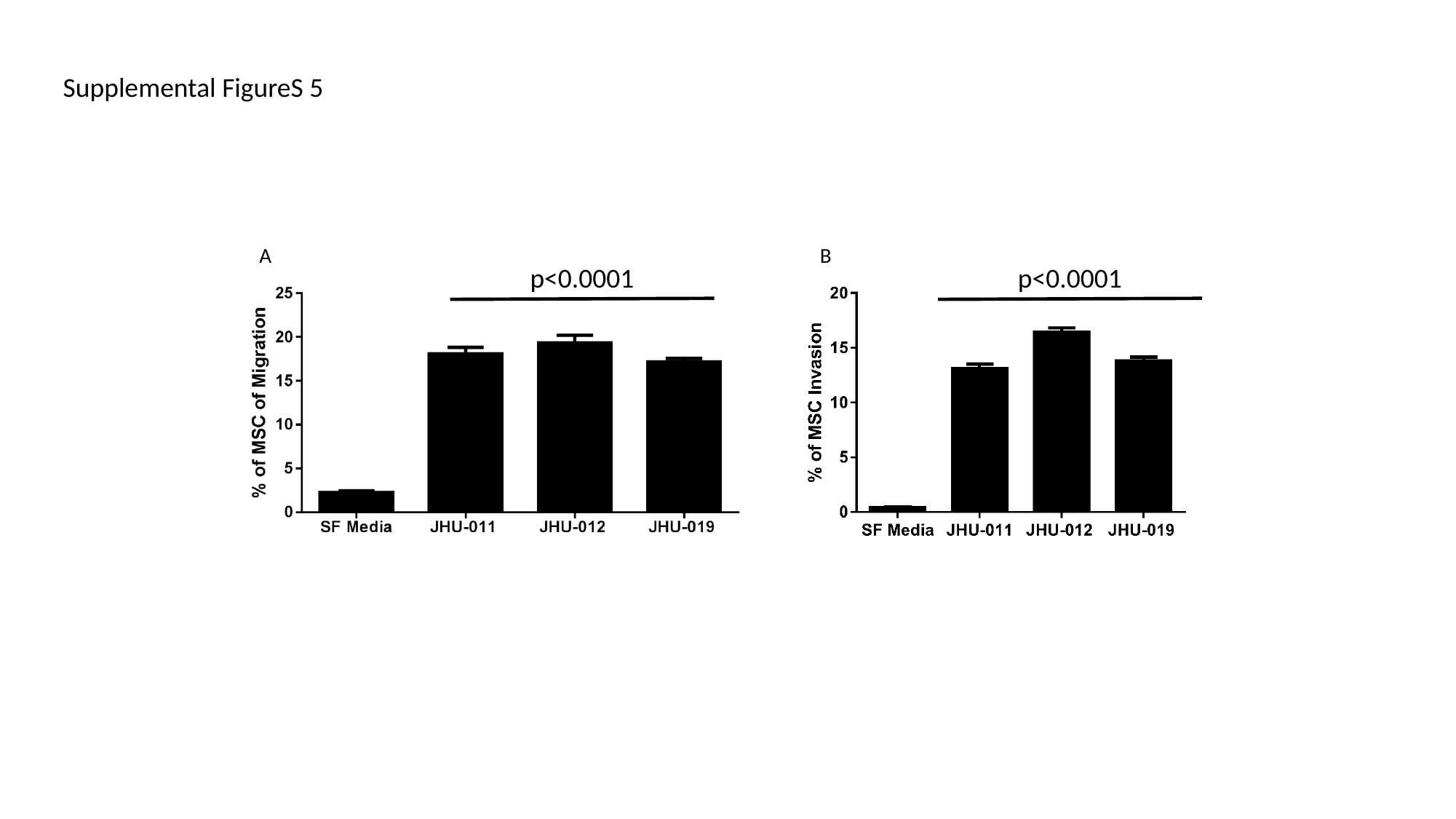

Supplemental FigureS 5
A
B
p<0.0001
p<0.0001

Supplement: Supplementary file 4 — Additional file 4: Figure S5. Conditioned media from 3 well characterized OPSCC cells lines (JHU-011, -012, and -019) caused significant migration and invasion of MSCs compared to serum free controls. Following 24-hour incubation with conditioned media from JHU-011, -012, and -019, a > sevenfold increase in MSC migration was observed when compared to serum free controls (A). MSCs were also observed to have a significant increased capacity for invasion > tenfold compared to that caused by the serum free controls (B). [file 12967_2016_1091_MOESM4_ESM.pptx]

## Slide 1
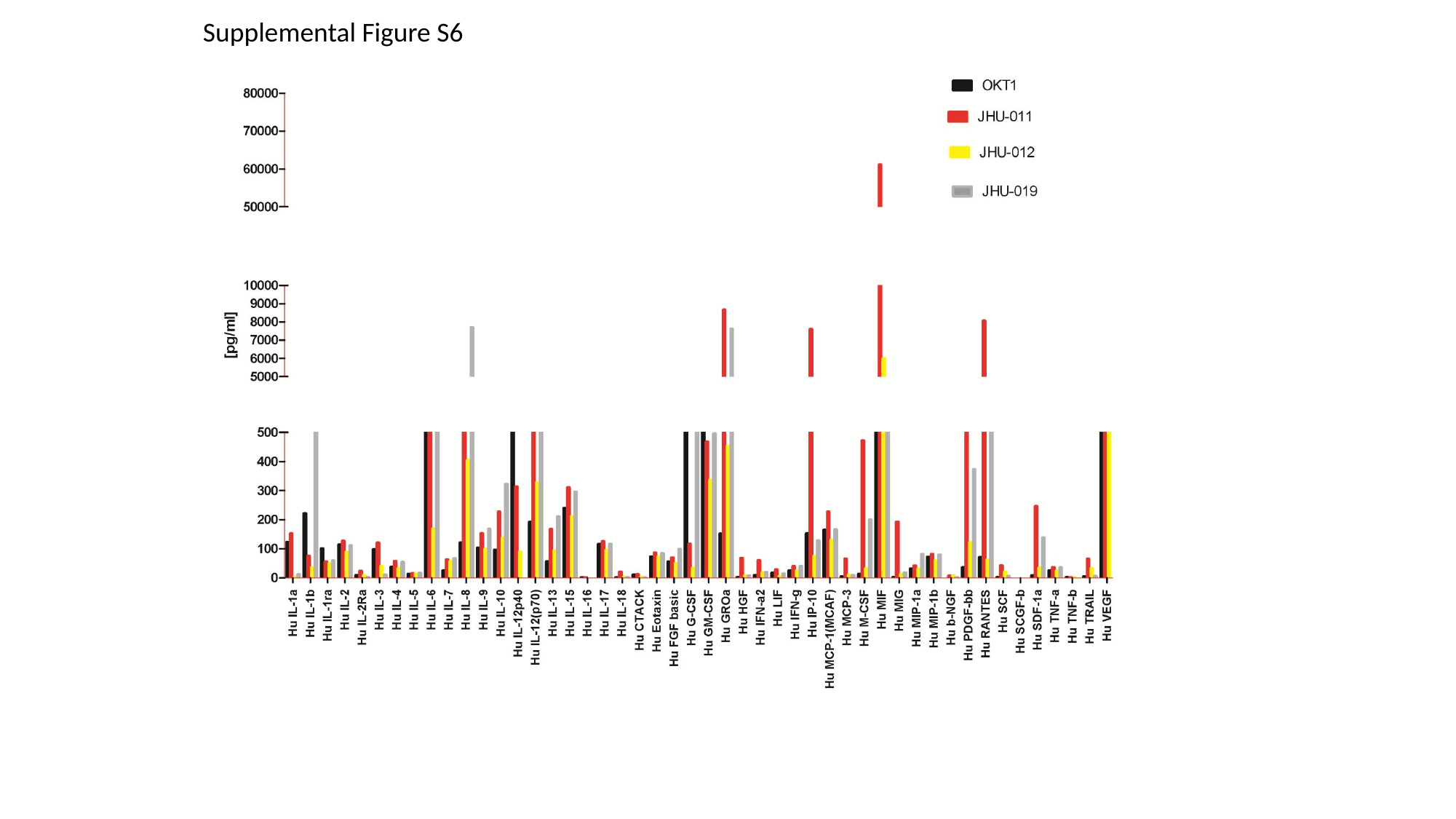

Supplemental Figure S6

Supplement: Supplementary file 5 — Additional file 5: Figure S6. Preliminary screening of condition media from OKT, JHU-011, -012, and -019 was performed using multi bead 21- and 27-plex assay from Bio-Rad. From this screening, SDF-1α, VEGF, Gro-α, IL-8, IL-6, and PDGF were found to be highly secreted compared to the conditioned media from OKT controls. Experiments were performed in duplicate for n = 3 observations. [file 12967_2016_1091_MOESM5_ESM.pptx]

## Slide 1
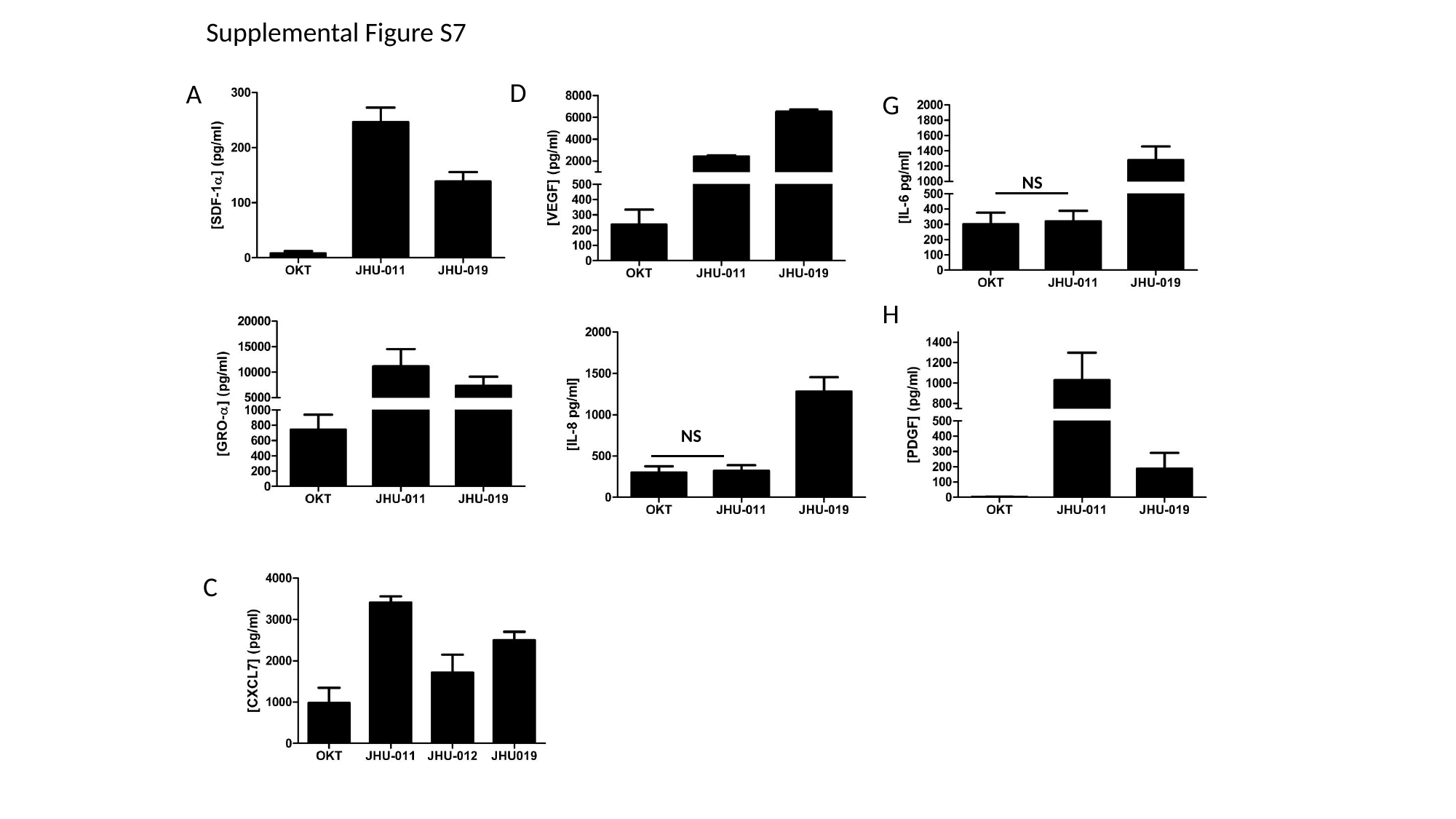

Supplemental Figure S7
D
A
G
NS
H
E
B
NS
F
C

Supplement: Supplementary file 6 — Additional file 6: Figure S7. Using the preliminary screening data, ELISA assays were conducted on SDF-1α, VEGF, Gro-α, IL-8, IL-6, and PDGF with the addition of CXCL7 to confirm the observations made in Additional file 1: Figure S6. [file 12967_2016_1091_MOESM6_ESM.pptx]

## Slide 1
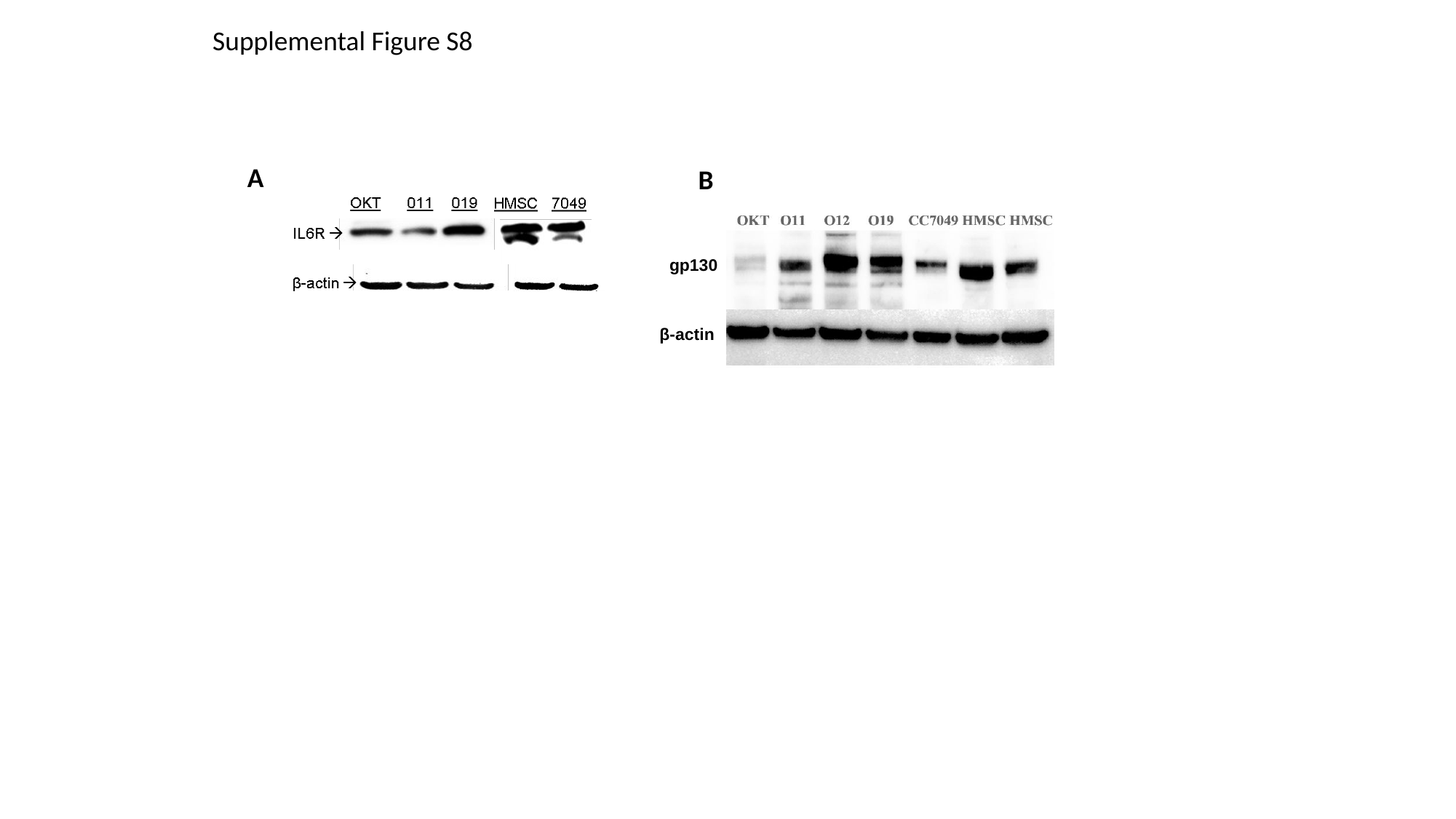

Supplemental Figure S8
A
B
gp130
β-actin

Supplement: Supplementary file 7 — Additional file 7: Figure S8. The IL-6 receptor and gp130 was expressed on the surface of OPSCC JHU-011, -012, -019 and on MSCs. Experiments were performed in triplicate. [file 12967_2016_1091_MOESM7_ESM.pptx]
